# Reduced contagious yawning in psychiatric disorders: a systematic review of potential social-cognitive mechanisms

**DOI:** 10.3389/fpsyt.2026.1879203

**Published:** 2026-10-05

**Authors:** Roni Boiangiu, Roni Peleg

**Affiliations:** 1 Department of Family Medicine and Siaal Research Center for Family Practice and Primary Care, The Haim Doron Division of Community Health, Faculty of Health Sciences, Ben-Gurion University of the Negev, Beer-Sheva, Israel; 2 Clalit Health Services, Beer-Sheva, Israel

**Keywords:** autism spectrum disorder, contagious yawning, human mirror neuron system, psychopathic traits, schizophrenia

## Abstract

**Introduction:**

Contagious yawning has been proposed to reflect multiple aspects of social cognition, including empathy, emotional contagion, self-processing, Theory of Mind (ToM), and action observation mechanisms such as the human mirror neuron system (hMNS). However, its underlying mechanisms remain debated, with evidence suggesting additional roles for social attention, gaze allocation, social motivation, and neurobiological factors. This systematic review examined evidence for reduced contagious yawning in Autism Spectrum Disorder (ASD), schizophrenia-spectrum disorders, and psychopathic traits, and evaluated potential underlying mechanisms.

**Methods:**

PubMed, PsycINFO, and Scopus were systematically searched for clinical and mechanistic studies of contagious yawning in psychiatric and healthy populations. Studies were screened using predefined eligibility criteria, and risk of bias was assessed using design-specific Joanna Briggs Institute (JBI) critical appraisal tools. Twenty-three studies met the inclusion criteria.

**Results:**

Reduced contagious yawning was reported in ASD, psychopathic traits, and schizophrenia-spectrum disorders, although findings varied across conditions. In ASD, reduced contagious yawning was associated with social attention, gaze allocation, oxytocin-related processes, and broader social-cognitive differences. In psychopathic traits, evidence more consistently implicated affective-interpersonal traits and reduced emotional responsiveness. Evidence for schizophrenia-spectrum disorders was more limited, with proposed mechanisms including impairments in social cognition, ToM, negative symptoms, and possible medication effects. Neuroimaging and electrophysiological findings implicate brain systems involved in social cognition, self-processing, and action observation, although the specific role of the hMNS remains uncertain.

**Discussion:**

Reduced contagious yawning may reflect altered social-cognitive processing rather than empathy alone, with contributing mechanisms differing across psychiatric conditions. Interpretation is limited by small sample sizes, methodological heterogeneity, and variation in study design. Further well-powered studies are needed to clarify its neurobiological basis and clinical significance.

## 1 Introduction

Spontaneous yawning is an evolutionarily conserved physiological behavior observed across many mammalian and vertebrate species. Although its precise function remains debated, proposed roles include regulation of arousal and vigilance, non-verbal social communication, and, more controversially, respiratory modulation and brain thermoregulation (1). Yawning is regulated by a complex neuroanatomical network involving the hypothalamus, particularly the paraventricular nucleus (PVN), which is influenced by neurotransmitter systems including oxytocinergic, dopaminergic, and nitric oxide pathways. These signals are integrated with brainstem mechanisms and modulated by limbic and cortical structures to produce the coordinated yawning response (1, 2).

Contagious yawning refers to the elicitation of yawning following perception or mental representation of another individual’s yawn, in contrast to spontaneous yawning, which is primarily associated with internal physiological states. Contagious yawning can be triggered by multiple social cues, including observing a yawning face, hearing a yawn, reading about yawning, or thinking about yawning (3). Experimental studies have demonstrated that dynamic visual presentations of yawning faces elicit more contagious yawning responses than control facial expressions, supporting the specificity of yawning as a socially relevant stimulus (4). Furthermore, the phenomenon does not appear to depend on a single facial feature or viewing angle, suggesting involvement of broader social perception mechanisms rather than isolated stimulus detection (4).

Several mechanisms have been proposed to explain individual differences in contagious yawning susceptibility. Early theories emphasized the relationship between contagious yawning and empathy, emotional contagion, and social bonding, proposing that these processes may contribute to the ability to resonate with another individual’s internal state (5, 6).

Other theories have proposed that contagious yawning may involve broader forms of social-cognitive processing, including self-processing, mental state attribution, and Theory of Mind (ToM). ToM reflects the ability to attribute mental states to oneself and others, including inferring others’ desires, thoughts, and beliefs in order to predict behavior (7). From an evolutionary perspective, self-awareness has been proposed as an important component of complex social cognition, enabling individuals to distinguish between self and others and supporting more advanced representations of social interactions (8). Consistent with this framework, Platek et al. (9) demonstrated associations between contagious yawning, self-face recognition, and ToM performance, suggesting a potential relationship between contagious yawning and processes involved in self-other distinction. However, systematic reviews have concluded that the association between contagious yawning and empathy remains inconsistent, indicating that multiple cognitive and neurobiological mechanisms may contribute to this phenomenon (10, 11).

Although empathy has traditionally been regarded as one of the principal mechanisms underlying contagious yawning, contemporary models of social cognition conceptualize empathy as a multidimensional construct comprising both affective and cognitive components. Cognitive empathy overlaps substantially with processes such as mental state attribution and Theory of Mind (ToM), whereas affective empathy is more closely related to emotional contagion and emotional responsiveness. These constructs should therefore not be viewed as mutually exclusive explanations but rather as complementary components of broader social-cognitive functioning. Accordingly, the present review evaluates contagious yawning within a multidimensional framework encompassing empathy, social attention, emotional contagion, mental state attribution, and action observation, rather than assuming a single underlying mechanism.

Neuroimaging and electrophysiological studies have further investigated the mechanisms underlying contagious yawning. Mirror neurons were originally described as neurons activated during both action execution and observation, and this system has been proposed to contribute to understanding others’ actions and social cognition (12, 13). Several neuroimaging and electrophysiological studies have identified activation of brain regions involved in social cognition during contagious yawning, including regions implicated in mentalization, self-processing, and neural systems proposed to overlap with the human mirror neuron system (hMNS) (5, 14–16). Alongside the hMNS, the default mode network (DMN), a large-scale brain network including regions such as the ventromedial prefrontal cortex (17) and posterior cingulate cortex, has been implicated in internally directed cognition, self-processing, and ToM (17, 18). Given that contagious yawning has been associated with processes involving mentalization and self-other distinction, the DMN has been proposed as a potential neural framework that may interact with action observation networks in supporting this complex social-cognitive behavior.

However, the contribution of the hMNS to contagious yawning and its relationship with empathy remain debated, and contagious yawning is likely influenced by multiple interacting processes rather than a single neural mechanism.

In the present systematic review, we focused on reduced contagious yawning in Autism Spectrum Disorder (ASD), schizophrenia-spectrum disorders, and psychopathy. These disorders were selected because they represent distinct patterns of social-cognitive differences and impairments. ASD is characterized by differences in social attention, communication, and gaze allocation, psychopathy is primarily associated with reduced affective empathy and emotional responsiveness, whereas schizophrenia-spectrum disorders involve impairments in ToM, social cognition, and negative symptoms. Examining these conditions together may provide insight into whether reduced contagious yawning reflects a shared alteration in social-cognitive processing or disorder-specific mechanisms.

Because relatively few studies have examined contagious yawning in clinically diagnosed psychiatric populations, the present review also includes investigations of trait-level characteristics, including psychopathic and schizotypal traits. Although findings from trait-based studies cannot be considered equivalent to observations in clinical populations, they provide complementary insight into dimensional variation in social-cognitive functioning and may help identify mechanisms that extend across traditional diagnostic boundaries.

## 2 Methods

### 2.1 Study design

The present systematic review aimed to synthesize the available evidence regarding contagious yawning across psychiatric conditions and to examine the social-cognitive and neurobiological mechanisms proposed to underlie this phenomenon. In addition, the methodological quality of the included studies was assessed to facilitate interpretation of the evidence, identify areas of convergence and inconsistency, and highlight priorities for future research.

The review was performed and reported in accordance with the Preferred Reporting Items for Systematic Reviews and Meta-Analyses (PRISMA) 2020 Statement.

The review question was formulated according to the PICOS framework:

- Population (P): Individuals diagnosed with Autism Spectrum Disorder (ASD), schizophrenia-spectrum disorders, psychopathy or psychopathic traits, as well as healthy participants included in mechanistic studies of contagious yawning.
- Intervention/Exposure (I): Exposure to contagious yawning paradigms, including visual, auditory, or experimentally induced yawning stimuli.
- Comparator (C): Healthy control participants or comparison groups with lower levels of psychiatric symptoms or psychopathic traits.
- Outcomes (O): Contagious yawning susceptibility and associated cognitive, behavioral, and neurobiological mechanisms proposed to contribute to individual differences or reductions in contagious yawning.
- Study Design (S): Analytical cross-sectional, quasi-experimental, and randomized controlled studies, including experimental neuroimaging and electrophysiological investigations.

A review protocol was not prospectively registered.

Because the primary aim of the review was to examine both reduced contagious yawning in psychiatric conditions and the potential mechanisms underlying this phenomenon, studies were considered eligible if they either examined psychiatric populations or clinically relevant trait dimensions, or investigated cognitive, behavioral, or neurobiological mechanisms of contagious yawning in healthy populations. Mechanistic studies conducted in healthy populations were therefore included to provide contextual evidence regarding potential mechanisms, but were not interpreted as evidence of psychiatric-group differences.

### 2.2 Literature search strategy

A comprehensive literature search was conducted in March 2026 using three electronic databases: PubMed, Scopus, and PsycINFO.

The search strategy combined controlled vocabulary terms (Medical Subject Headings [MeSH], where applicable) with free-text keywords related to contagious yawning and the psychiatric disorders of interest. Search terms included combinations of *contagious yawning*, *yawn contagion*, *schizophrenia*, *psychotic disorders*, *autism spectrum disorder*, *psychopathy*, *antisocial personality disorder*, and *callous-unemotional traits*.

The search strategy was adapted for each database to accommodate its specific indexing structure, controlled vocabulary, and search functionality; therefore, the search syntax and fields varied across databases. The complete database-specific search strategies are provided in **Supplementary File 1**.

No restrictions were placed on publication year. Only peer-reviewed studies published in English were eligible for inclusion.

To maximize the completeness of the search, reference lists of included studies were screened to identify additional relevant publications. Studies identified through this process that did not meet eligibility criteria were not included in the systematic synthesis but were used only as background references where appropriate.

### 2.3 Eligibility criteria

Studies were included if they met all of the following criteria:

1. Original peer-reviewed research articles.
2. Human participants.
3. Investigation of contagious yawning in psychiatric populations of interest (ASD, schizophrenia-spectrum disorders, psychopathy/psychopathic traits) or healthy populations when examining cognitive or neurobiological mechanisms relevant to contagious yawning.
4. Evaluation of behavioral, cognitive, or neurobiological aspects of contagious yawning.
5. Reporting of original quantitative data.

Studies were excluded if they met one or more of the following criteria:

1. Animal studies.
2. Narrative reviews, systematic reviews, meta-analyses, editorials, conference abstracts, letters, dissertations, or case reports.
3. Studies evaluating spontaneous yawning without assessment of contagious yawning.
4. Articles published in languages other than English.
5. Studies lacking sufficient methodological information or original outcome data.

### 2.4 Study selection

All records retrieved from the electronic database searches were compiled into a single dataset. Duplicate records were identified and removed manually before screening.

Titles and abstracts were initially screened by the first author (RB) according to the predefined eligibility criteria. Studies considered potentially eligible subsequently underwent full-text review independently by both authors. Disagreements regarding study eligibility were resolved through discussion until consensus was achieved.

The study selection process is summarized in the PRISMA flow diagram (**Figure 1**).

**Figure 1:**
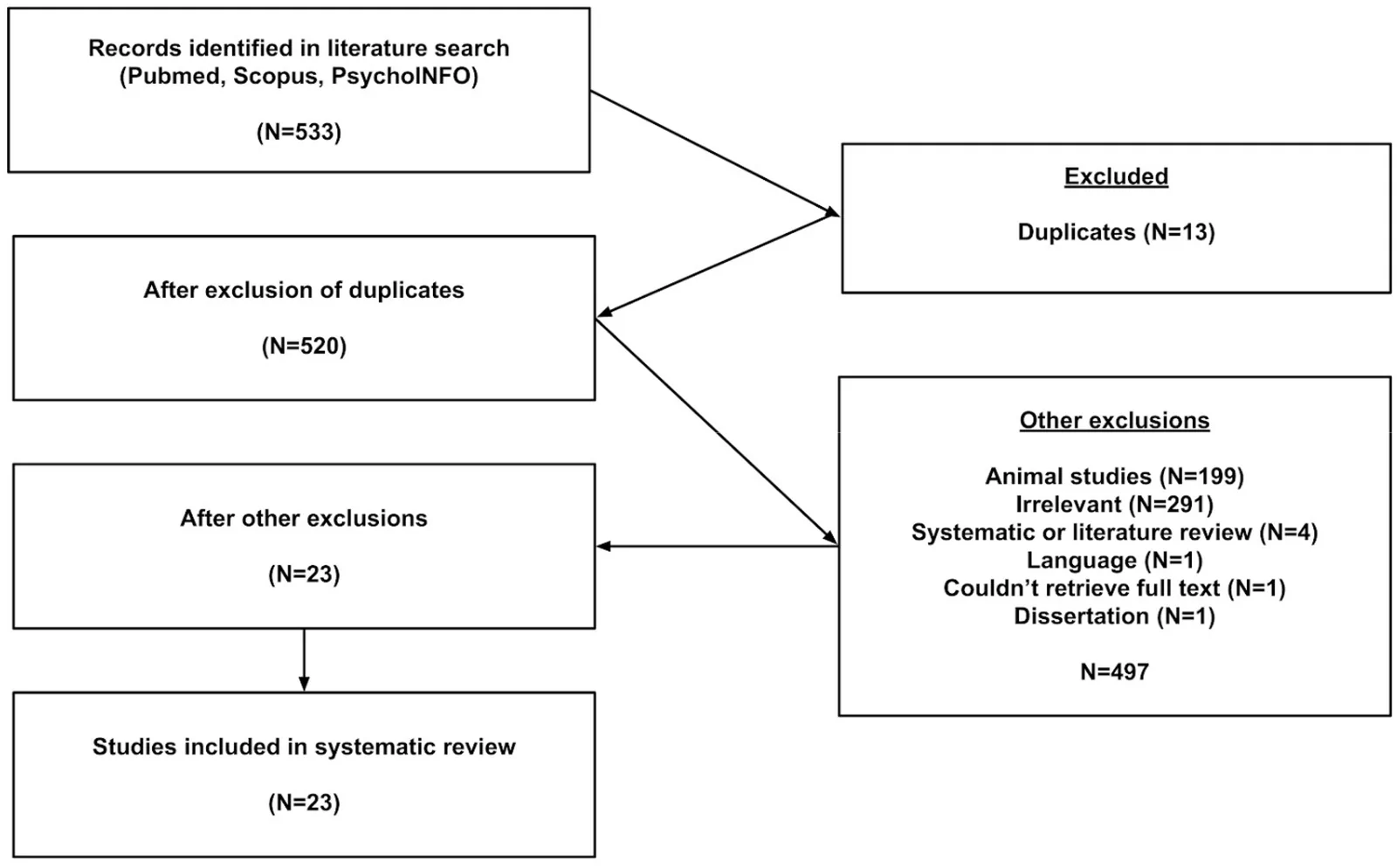
Flowchart of the review process.

### 2.5 Data extraction

Data extraction was performed independently by both reviewers using a predefined data extraction framework developed before the review commenced.

For each eligible study, the following information was extracted:

- first author;
- year of publication;
- study design;
- sample size;
- participant characteristics;
- psychiatric diagnosis or clinical population;
- contagious yawning paradigm;
- primary outcome measures;
- principal findings;
- proposed cognitive or neurobiological mechanisms.

Following independent extraction, both reviewers compared the extracted information, and any discrepancies were resolved through discussion until consensus was achieved.

### 2.6 Quality assessment

Methodological quality and risk of bias were assessed using the Joanna Briggs Institute (JBI) critical appraisal tool appropriate to the design of each included study (19–21). Separate JBI checklists were applied to analytical cross-sectional, quasi-experimental, and randomized controlled studies. Each appraisal item was rated as yes (Y), no (N), unclear (U), or not applicable (NA). The assessment considered the clarity of participant selection, description of the study population and setting, validity and reliability of exposure and outcome measures, identification and management of potential confounding factors, and appropriateness of statistical analyses. For experimental studies, additional domains addressed the presence and comparability of control groups, consistency of procedures across comparison conditions, outcome assessment, follow-up, and, for randomized studies, randomization, allocation concealment, and blinding. The appraisal was used to characterize methodological strengths and limitations across the included studies and to inform the interpretation of the findings. Studies were not excluded solely on the basis of their methodological appraisal. Detailed item-level appraisal results are presented in **Supplementary Tables 1**-**3**.

### 2.7 Data synthesis

Because of substantial methodological heterogeneity across the included studies, including differences in study populations, psychiatric diagnoses, experimental paradigms, outcome measures, and reported outcomes, a quantitative meta-analysis was considered inappropriate.

Instead, a narrative synthesis was conducted. Studies were initially organized according to psychiatric disorder (Autism Spectrum Disorder, psychopathic traits, and schizophrenia-spectrum disorders). Subsequently, findings were critically synthesized according to the mechanisms proposed to contribute to contagious yawning susceptibility, including empathy, emotional contagion, ToM, social attention, gaze allocation, oxytocin-related mechanisms, and neural systems involved in social cognition. Areas of agreement, conflicting evidence, and methodological limitations were identified to provide an integrated interpretation of the current literature.

## 3 Results

### 3.1 Study selection

The electronic database search identified 533 records across PubMed, Scopus, and PsycINFO. Following removal of 13 duplicate records, 520 unique records remained for title and abstract screening. During the initial screening phase, 491 records were excluded because they did not meet the predefined eligibility criteria.

During full-text assessment, six additional records were excluded because they were review articles or systematic reviews (n=4), a dissertation (n=1), or because the full text could not be obtained (n=1).

Following full-text evaluation, 23 studies met all inclusion criteria and were included in the final systematic review. The study selection process is summarized in the PRISMA flow diagram (**Figure 1**).

The articles included in the review are shown in **Table 1**. The year of publication ranged from 2003 to 2023. The characteristics of the articles that were included in the review are shown in **Table 2**.

**Table 1:** Studies included in the systematic review.

Lead author	Paper	Publication year	Reason for inclusion
Poole, K	Social cognitive correlates of contagious yawning and smiling.	2023	Social attachment and behavior were associated with contagious yawning.
Helt, M.S.	Patterns of contagious yawning and itching differ among adults with autistic traits vs. psychopathic traits.	2021	An association was shown between psychopathic traits and reduced yawning, apparently mediated by eye movements.
Helt, M.S.	Contagious itching is heightened in children with autism spectrum disorders.	2021	Absence of contagious yawning in children on the spectrum. In the control TD (typically developing) group, contagious yawning was associated with emotional empathy.
Gallup, A.C.	People that score high on psychopathic traits are less likely to yawn contagiously	2021	People with psychopathic traits yawn less than controls. A reverse association was seen between psychopathic traits and contagious yawning.
Norscia, I.	Auditory contagious yawning is highest between friends and family members: support for the emotional bias hypothesis,	2020	The social connection between the participants was a significant predictor for contagious yawning events. The strongest prevalence was among friends and family, providing support for the emotional bias hypothesis.
Helt, M.S.	Emotional contagion in children with autism spectrum disorder varies with stimulus familiarity and task instructions.	2020	Children with ASD (autism spectrum disorders) yawned contagiously less compared to TD. This difference was demonstrated when they were asked to focus on a visual stimulus or in reaction to their parents.
Mariscal, M.G.	Blood oxytocin concentration positively predicts contagious yawning behavior in children with autistic spectrum disorder	2019	In terms of contagious yawning there was no difference between ASD and controls. There was a correlation between the concentration of oxytocin and contagious yawning.
Franzen, A.	Contagious yawning, empathy, and their relation to prosocial behavior.	2018	Contagious yawning was associated with empathy trait.
Chan, M.H.M.	Yawning detection sensitivity and yawning contagion.	2017	There was no correlation between autistic traits and contagious yawning.
Rundle, B.K.	Contagious yawning and psychopathy.	2015	Among psychopathic traits, reduced emotional responsiveness was associated with absence of contagious yawning. The amplitude of the startle reaction to a stimulus helped predict contagious yawning.
Bartholomew, A.J.	Individual variation in contagious yawning susceptibility is highly stable and largely unexplained by empathy or other known factors.	2014	There was no association between empathy, emotional attachment, sleepiness, or circadian rhythm and contagious yawning.
Haker, H.	Mirror neuron activity during contagious yawning: an fMRI study.	2013	The fMRI (functional MRI) mapping showed activation of Brodman area 9 in the right inferior frontal gyrus and an association between this area and the human mirror neuron system for tasks requiring mentalization.
Cooper, N.R.	Bridging a yawning chasm: EEG investigations into the debate concerning the role of the human mirror neuron in contagious yawning.	2012	This EEG (electroencephalogram) study demonstrated activity that characterizes the human mirror neuron system while observing contagious yawning. This activity was correlated with empathy.
Norscia, I.	Yawn contagion and empathy in Homo Sapiens.	2011	Social bonding could predict the prevalence, frequency, and latency of contagious yawning. Contagious yawning derived primarily from the emotional proximity between people. The contagion rate was higher with exposure to family and friends.
Helt, M.S.	Contagious yawning in autistic and typical development.	2010	The autistic group had reduced contagious yawning compared to controls.
Senju, A.	Brief report. Does eye contact induce contagious yawning in children with autism spectrum disorder.	2009	No difference was found in contagious yawning between autistic spectrum disorder and normal development when asked to focus their look on the eyes who were presented as a visual stimulus.
Arnott, S.R.	An investigation of auditory contagious yawning.	2009	An fMRI study that demonstrated increased activity in the right posterior inferior frontal gyrus in reaction to the sound of yawning, and with increased need to yawn, correlated with empathy.
Haker, H.	Empathy in schizophrenia: impaired resonance.	2009	Schizophrenic patients manifested less contagious yawning and laughing. Contagion was not correlated with empathy.
Nahab, F.B.	Contagious yawning and the frontal lobe: an fMRI study.	2009	An fMRI study demonstrated activation of the ventromedial, para-frontal cortex while observing yawning.
Giganti, F.	Contagious and spontaneous yawning in autistic and typically developing children.	2009	There was a significant impairment in the effect of yawning in children with ASD. There was no difference in the frequency and distribution of spontaneous yawning during the day compared to controls.
Senju, A.	Absence of contagious yawning in children with autistic spectrum disorder.	2007	The control group yawned more in response to yawning videos, compared to children with ASD.
Platek, S.M.	Contagious yawning and the brain.	2005	An fMRI study that demonstrated that observing a yawning person activated the posterior cingulate region and the precuneus.
Platek, S.M.	Contagious yawning: the role of self-awareness and mental state attribution.	2003	Schizotypal traits had a negative correlation with contagious yawning. There was a positive correlation between contagious yawning and TOM (Theory of mind) and facial recognition.

TD, typically developing; ASD, autism spectrum disorders; fMRI, functional MRI; EEG, electroencephalogram; ToM, Theory of mind.

**Table 2:** Demographic and clinical characteristics of included studies.

Author (Citation number)	Sample size (N)	Participant type	Age range	Mean age (SD)	Gender split	Key focus group
Gallup et al. (34)	458	Community	18–58 yrs	20.42 (SD 4.10)	329 F/124 M/5 Other	Psychopathic Traits (PPI-R)
Helt et al. (29)	97	College Students	18.75–24.58 yrs	21.48 (SD 1.93)	50 F/47 M	Autistic & Psychopathic Traits
Helt et al. (37)	161	ASD (autism spectrum disorders) vs. TD (typically developing)	5.41–15.58 yrs	11.48 (ASD)/11.91 (TD)	19 F/142 M	ASD (55) vs TD (106)
Helt et al. (31)	120	ASD vs. TD	5–17 yrs	10.8 (ASD)/8.2 (TD)	6 F/114 M	Familiarity & Task Instructions
Norscia et al. (24)	153	Community	18–77 yrs	*Categorized by group*	69 F/84 M	Social Bonding/Auditory Yawn
Mariscal et al. (33)	64	Children	6–12 yrs	*Not reported*	15 F/49 M	ASD (35) vs. TD (32)/Oxytocin
Franzen & Mader (22)	504	College Students	19–34 yrs	~23.7 (SD ~3.0)	343 F/161 M	Empathy/Prosociality (2 Studies)
Chan & Tseng (32)	41	Healthy Adults	19–26 yrs	21.3 (SD 1.89)	21 F/20 M	Eye-tracking/ASD Traits
Rundle et al. (35)	135	College Students	*Not reported*	18.89 (SD 1.13)	78 F/57 M	Psychopathic Traits (PPI-R)
Bartholomew (10)	328	Healthy Volunteers	18–83 yrs	32.19 (SD 15.06)	220 F/108 M	Age, Empathy, Sleepiness
Haker et al. (5)	11	Healthy Adults	21–55 yrs	31.5 (*NR -* - Not Reported by the authors in the text)	6 F/5 M	fMRI (functional MRI)/Mirror Neuron System
Cooper et al. (15)	72	General Population	18–44 yrs	~23.5 (SD 5.0)	39 F/33 M	EEG (electroencephalogram)/Mu Suppression (3 Studies)
Norscia & Palagi (6)	109	Community	>16 yrs	*Not reported*	56 F/53 M	Naturalistic/Social Bonding
Helt et al. (27)	204	TD & ASD Children	1–16 yrs	*Varies by study*	74 F/130 M	Developmental Trajectory (2 Studies)
Nahab et al. (25)	18	Healthy Adults	18–48 yrs	29.5 (SD 9.3)	9 F/9 M	fMRI/Ventromedial PFC
Senju et al. (30)	62	ASD vs. TD	7–17 yrs	~12.0 (SD ~2.9)	23 F/39 M	ASD (33) vs. TD (33)/Eye-fixation
Arnott et al. (14)	10	Healthy Adults	*Not reported*	27.7 (SD 4.2)	6 F/4 M	Auditory fMRI/Posterior IFG
Haker & Rössler (36)	88	Schizophrenia vs TD	*Not reported*	~36.8 (SD ~10.5)	10 F/78 M	Schizophrenia (43) vs. TD (45)
Giganti & Ziello (26)	27	HFA/LFA/TD	10–15 yrs	12.2 (*NR*)	11 F/16 M	HFA (high-functioning autistic children) (7), LFA (low-functioning autistic children) (10), TD (10)
Senju et al. (28)	49	ASD vs. TD	7–15.9 yrs	11.3 (SD ~2.2)	15 F/34 M	ASD (26) vs. TD (27)
Platek et al. (16)	10	College Students	*Not reported*	21.5 (*NR*)	6 F/4 M	fMRI/Precuneus & Post. Cingulate
Platek et al. (9)	65	College Students	*Not reported*	19.8 (*NR*)	34 F/31 M	Schizotypal Traits/Theory of Mind
Poole & Henderson (23)	154	College Students	*Not reported*	21.31 (SD 2.57)	121 F/28 M/5 NB (Non binary)	Empathy/Behavioral

ASD, autism spectrum disorders; TD, typically developing; NR, Not reported by the authors in the text; fMRI, functional MRI; EEG, electroencephalogram; HFA, high-functioning autisticchildren; LFA, low-functioning autistic children; NB, non binary.

### 3.2 Characteristics of included studies

The 23 included publications employed diverse methodologies, including analytical cross-sectional, quasi-experimental, and randomized designs, as well as experimental investigations using functional magnetic resonance imaging (fMRI) and electroencephalography (EEG).

Of the 23 included studies, seven examined psychiatric populations or clinically relevant trait dimensions, including Autism Spectrum Disorder (ASD), psychopathic traits, schizophrenia, and schizotypal traits. The remaining 16 studies examined cognitive, behavioral, or neurobiological mechanisms associated with contagious yawning in healthy populations. These mechanistic studies were included to contextualize potential processes underlying contagious yawning and were not considered evidence of psychiatric-group differences. This distinction was maintained throughout the synthesis and interpretation.

Contagious yawning was assessed using a broad range of experimental paradigms, including visual and auditory yawning stimuli, dynamic and static presentations, eye-fixation manipulations, familiar versus unfamiliar actors, and naturalistic observation protocols. Considerable variation was also observed in the operational definition of contagious yawning, outcome measures, and stimulus exposure. Overall, this methodological heterogeneity represents an important source of variability across the literature and should be considered when interpreting differences between studies. Because different paradigms likely engage partially distinct aspects of social cognition, methodological differences may account for some of the inconsistencies observed across findings.

### 3.3 Cognitive and neurobiological mechanisms associated with contagious yawning

Nine studies investigated cognitive and neurobiological variables associated with contagious yawning susceptibility.

Several behavioral studies examined associations between contagious yawning and social-affective characteristics (6, 22, 23). Franzen reported associations between contagious yawning responses and measures related to emotional attachment, interpersonal relationships, or prosocial characteristics. Two studies (6, 24) reported higher rates of contagious yawning among individuals with closer social relationships, including friends and family members, compared with unfamiliar individuals.

In contrast, other studies did not identify significant associations between contagious yawning and empathy-related measures. Bartholomew and Cirulli (10) examined individual differences in contagious yawning susceptibility and reported that these differences were not explained by empathy scores, emotional attachment, sleepiness, or circadian factors. Similarly, Massen and Gallup (11) reviewed the available literature and concluded that current quantitative evidence does not establish contagious yawning as a specific measure of empathy.

Neuroimaging studies investigated neural responses during exposure to yawning stimuli in paradigms designed to examine contagious yawning. Studies using fMRI reported activation in regions involved in social cognition, self-processing, and action observation, including the inferior frontal gyrus, posterior cingulate cortex, precuneus, and ventromedial prefrontal cortex (5, 14, 16, 25). These findings reflect neural responses observed during exposure to yawning stimuli and should not be interpreted as demonstrating that these regions directly cause the behavioral expression of contagious yawning.

Cooper et al. (15) examined electrophysiological responses during observation of yawning stimuli and reported mu suppression in sensorimotor regions.

### 3.4 Contagious yawning in autism spectrum disorder

Several studies compared contagious yawning responses between individuals with ASD and typically developing (TD) control participants and reported reduced contagious yawning frequencies in ASD cohorts (26–28).

Giganti and Ziello (26) reported that group differences were specific to contagious yawning, as spontaneous yawning frequencies did not differ between ASD and TD groups.

Helt et al. (29) examined the relationship between contagious yawning and emotional empathy and reported a positive association between contagious yawning and emotional empathy scores in the TD group; this association was not observed in the ASD group.

Other studies investigated the influence of social attention and gaze allocation on contagious yawning responses. Senju et al. (30) reported that when participants with ASD were instructed to focus their gaze on socially relevant facial regions, differences in contagious yawning between ASD and TD groups were reduced. Similarly, Helt et al. (31) reported that differences between groups varied according to stimulus characteristics, including the use of familiar versus unfamiliar yawning stimuli.

Other studies further suggest that contagious yawning may not be directly related to autistic traits themselves, but rather to specific mechanisms involved in processing social cues. Chan and Tseng (32) examined autistic traits in a non-clinical student population and found no association between autistic trait scores and contagious yawning susceptibility. The authors suggested that individuals who are more susceptible to contagious yawning may show increased sensitivity to the perceptual processing of yawning cues rather than reflecting sensitivity to broader emotional information conveyed by others. In addition to cognitive and attentional mechanisms, biological factors may also contribute to individual differences in contagious yawning responses. Mariscal et al. (33) examined oxytocin concentrations in children with ASD and reported a positive association between blood oxytocin levels and contagious yawning behavior.

### 3.5 Contagious yawning and psychopathic traits

Three studies examined the relationship between contagious yawning and psychopathic traits (29, 34, 35). All three studies investigated community or student samples using psychopathic trait measures rather than clinically diagnosed psychopathy.

Across these studies, higher psychopathic trait scores were associated with reduced contagious yawning susceptibility. Rundle (35) reported negative associations between contagious yawning and affective-interpersonal dimensions of psychopathy, including callousness and reduced emotional responsiveness.

Additionally, Rundle et al. examined physiological responses and reported that startle response amplitude was associated with contagious yawning susceptibility (35).

### 3.6 Contagious yawning in schizophrenia-spectrum conditions

Two studies provided data relevant to schizophrenia-spectrum conditions.

Haker and Rössler (36) examined individuals with clinically diagnosed schizophrenia and reported reduced contagious yawning and contagious laughing responses compared with healthy control participants.

Platek et al. (9) investigated schizotypal traits in a non-clinical sample of college students. The authors reported a negative association between schizotypal trait scores and contagious yawning susceptibility. Additionally, performance on ToM and self-face recognition tasks was positively associated with contagious yawning susceptibility.

Because Platek et al. (9) examined schizotypal traits rather than schizophrenia, these findings provide information regarding schizophrenia-spectrum characteristics but cannot be directly generalized to individuals with clinical schizophrenia.

Accordingly, evidence from schizotypal traits was considered complementary to, rather than equivalent to, evidence from clinically diagnosed schizophrenia.

### 3.7 Risk of bias and methodological limitations

The methodological appraisal indicated that the included studies were generally adequately reported with respect to participant selection, study procedures, outcome assessment, and statistical analysis, although methodological characteristics varied across study designs. Most studies used experimental or observational paradigms that allowed contagious yawning to be examined in relation to participant characteristics, stimulus conditions, or clinical and trait-related characteristics. Several studies included comparison groups or controlled stimulus conditions, providing a basis for examining differences in contagious yawning across populations and experimental contexts. Some methodological limitations were identified in individual studies, including the use of within-subject control conditions rather than independent control groups in several experimental studies, differences in participant characteristics between comparison groups, and limited reporting of outcome-measurement reliability in some studies. A small number of studies also involved relatively small samples or multiple statistical comparisons, resulting in some uncertainty regarding the robustness of individual findings. The randomized component of Franzen et al. (22) provided additional methodological control through random allocation, although allocation concealment and blinding were not fully documented. These limitations varied across studies and did not uniformly affect the evidence base. Overall, the appraisal supports consideration of the included studies as a heterogeneous body of evidence, while indicating that findings should be interpreted with appropriate consideration of differences in study design and methodological rigor. Detailed JBI appraisal results are provided in **Supplementary Tables 1**-**3**.

## 4 Discussion

This systematic review synthesized the available evidence on contagious yawning across Autism Spectrum Disorder (ASD), psychopathic traits, and schizophrenia-spectrum disorders to evaluate the social-cognitive mechanisms that may underlie reduced contagious yawning. The evidence base included both studies directly examining psychiatric or clinically relevant trait populations and mechanistic studies conducted in non-clinical samples, which were considered as complementary evidence regarding potential underlying processes rather than as evidence of psychiatric-group differences. Across the reviewed literature, reduced contagious yawning emerged as a relatively consistent behavioral finding. However, the evidence does not support a single explanatory mechanism. Rather, the collective findings indicate that contagious yawning is best understood as a multidimensional social-cognitive phenomenon involving the interaction of social attention, emotional responsiveness, interpersonal familiarity, mental state attribution, and action observation. Importantly, the evidence supporting these mechanisms is not equally robust. Behavioral studies consistently implicate broader social-cognitive functioning, whereas evidence for specific neurobiological mechanisms remains comparatively limited. Consequently, contagious yawning should be interpreted as a potential behavioral marker of social-cognitive functioning rather than as a direct measure of empathy or any single cognitive process.

Another important observation is that inconsistencies across studies are more likely to reflect methodological heterogeneity than genuine theoretical disagreement. Included studies differed substantially in participant characteristics, diagnostic definitions, experimental paradigms, stimulus modality, familiarity of yawning stimuli, eye-fixation instructions, and outcome measures used to quantify contagious yawning. Because these paradigms engage partially distinct aspects of social cognition, methodological variation probably contributes to the variability of reported findings. Therefore, apparently conflicting results should be interpreted within the methodological context of each study rather than as evidence against particular theoretical models.

### 4.1 Disorder-specific mechanisms underlying reduced contagious yawning

The findings of this review suggest that although reduced contagious yawning has been observed across ASD, psychopathic traits, and schizophrenia-spectrum conditions, the mechanisms contributing to this phenomenon may differ substantially between disorders. These conditions are characterized by distinct patterns of social cognition, emotional processing, and interpersonal functioning, and therefore a single explanatory mechanism is unlikely to account for reduced contagious yawning across all populations.

#### 4.1.1 Autism spectrum disorder

Among the psychiatric conditions reviewed, ASD provides the strongest evidence linking reduced contagious yawning to alterations in social-cognitive functioning. Multiple independent studies consistently demonstrated reduced contagious yawning in autistic individuals compared with typically developing controls (26–28).

Importantly, however, the collective evidence indicates that reduced contagious yawning in ASD is unlikely to be explained by impaired empathy alone. Instead, the findings consistently support a multifactorial model in which social attention, gaze allocation, emotional responsiveness, and contextual factors interact to influence susceptibility to contagious yawning.

One of the most compelling findings concerns the role of visual attention. Senju et al. (30) demonstrated that directing children with ASD to fixate on the eye region substantially reduced the difference in contagious yawning between autistic and typically developing participants. Together with evidence of atypical gaze patterns in ASD (38), these findings suggest that diminished contagious yawning may result, at least in part, from reduced attention to socially relevant cues rather than an intrinsic inability to respond empathically. Similarly, Helt et al. (31) showed that contagious yawning varied according to stimulus familiarity and task instructions, further indicating that contextual and attentional factors significantly influence yawning susceptibility. Collectively, these experimental manipulations provide stronger support for attentional mechanisms than would be obtained from observational associations alone.

The distinction between contagious and spontaneous yawning provides additional support for this interpretation. Giganti and Ziello (26) reported that although children with ASD exhibited significantly reduced contagious yawning, spontaneous yawning frequency did not differ from that of typically developing controls. This finding argues against a general physiological impairment in yawning and instead suggests that the observed deficit is specific to socially elicited yawning, reinforcing the involvement of higher-order social-cognitive processes.

Nevertheless, the ASD literature also illustrates why contagious yawning should not be interpreted as a simple behavioral proxy for empathy. Mariscal et al. (33) found no overall difference in contagious yawning between autistic and typically developing children while reporting a positive association between oxytocin concentration and contagious yawning within the ASD group. Although these findings raise the possibility that neurobiological factors contribute to contagious yawning susceptibility, they are based on a single study and therefore provide only preliminary evidence. Similarly, while several studies have linked contagious yawning to emotional contagion or empathic processes, the collective literature suggests that these mechanisms operate alongside attentional and social-cognitive factors rather than independently explaining contagious yawning.

Overall, the evidence for ASD is both the most extensive and the most methodologically convincing among the psychiatric conditions reviewed. Multiple studies have replicated reduced contagious yawning using different experimental paradigms, and several have experimentally manipulated variables such as gaze direction and stimulus familiarity, strengthening confidence in the observed behavioral associations. At the same time, methodological differences in participant age, autism severity, and experimental design likely contribute to residual inconsistencies across studies. Consequently, current evidence supports reduced contagious yawning in ASD as a robust behavioral finding while favoring a multidimensional social-cognitive interpretation over explanations based solely on empathy deficits.

#### 4.1.2 Psychopathic traits

Compared with ASD, the evidence linking psychopathic traits to reduced contagious yawning is less extensive but relatively consistent. Across the reviewed studies, individuals with higher levels of psychopathic traits generally exhibited reduced susceptibility to contagious yawning (29, 34, 35), suggesting that this association is reproducible despite the smaller evidence base. However, because nearly all studies were conducted in non-clinical populations, confidence in the generalizability of these findings to individuals with clinically diagnosed psychopathy remains moderate.

Unlike the ASD literature, the findings in psychopathy provide less support for attentional explanations and instead point toward alterations in affective and interpersonal functioning. Rundle et al. (35) reported that psychopathic characteristics associated with reduced emotional responsiveness predicted lower contagious yawning susceptibility, while Gallup et al. (34), using one of the largest samples included in this review, similarly demonstrated an inverse relationship between psychopathic traits and contagious yawning. Helt et al. (29) further showed that individuals with elevated psychopathic traits displayed patterns of contagious yawning that differed from those associated with autistic traits, suggesting that superficially similar behavioral outcomes may arise through different underlying mechanisms.

Importantly, these findings argue against interpreting contagious yawning as a direct measure of empathy. Although psychopathy is characterized by impairments in affective empathy, aspects of cognitive social functioning, including mental state attribution, may remain relatively preserved (39). If contagious yawning reflected empathy alone, stronger and more consistent associations would be expected across psychopathy studies. Instead, the reviewed evidence suggests that contagious yawning is influenced by a broader constellation of social-cognitive processes, including emotional salience, interpersonal engagement, and social motivation. This interpretation is consistent with the broader conclusions of the present review, which indicate that empathy contributes to—but does not fully explain—contagious yawning.

Although the psychopathy studies were generally adequately reported, the evidence remains limited by the characteristics of the available samples. Most investigations examined psychopathic traits in community or university populations rather than clinically diagnosed psychopathy, limiting direct clinical interpretation. Consequently, while reduced contagious yawning appears to represent a reliable behavioral correlate of psychopathic traits, further research involving well-characterized clinical samples is required before disorder-specific conclusions can be drawn.

#### 4.1.3 Schizophrenia-spectrum disorders

Evidence regarding schizophrenia-spectrum disorders remains the most limited among the psychiatric conditions reviewed. This should not be interpreted as evidence against an association between schizophrenia-spectrum pathology and contagious yawning, but rather as reflecting the scarcity of available research. Only two studies met the inclusion criteria, one involving individuals with clinically diagnosed schizophrenia (36) and the other examining schizotypal personality traits in a non-clinical sample (9). Although both studies reported reduced contagious yawning, the small evidence base substantially limits confidence in mechanistic interpretations.

Haker and Rössler (36) found that individuals with schizophrenia exhibited reduced contagious yawning and contagious laughter compared with healthy controls. Notably, these reductions were not significantly associated with empathy measures, suggesting that diminished contagious yawning cannot be explained solely by impaired empathic functioning. Similarly, Platek et al. (9) reported that higher schizotypal traits were associated with reduced contagious yawning while also demonstrating relationships with Theory of Mind and facial recognition performance. Because schizotypal traits represent a recognized vulnerability phenotype for schizophrenia (41), these findings provide indirect support for the broader relevance of social-cognitive dysfunction within the schizophrenia spectrum. Nevertheless, results obtained in non-clinical populations should not be considered equivalent to findings in patients with schizophrenia.

Overall, the available evidence suggests that reduced contagious yawning in schizophrenia-spectrum disorders is more likely to reflect generalized disturbances in social cognition than deficits in any single psychological process. Schizophrenia is characterized by impairments in multiple domains, including Theory of Mind, emotion recognition, social perception, and interpersonal functioning (40), each of which could plausibly influence contagious yawning susceptibility. At present, however, the relative contribution of these mechanisms cannot be determined because of the limited number of available studies.

Interpretation of the schizophrenia literature is further complicated by methodological factors. Clinical samples differed with respect to illness duration, symptom severity, medication status, and cognitive functioning, variables that were not consistently controlled across studies. In addition, schizophrenia is frequently associated with abnormalities in eye movements and visual exploration (42), potentially influencing attention toward socially relevant cues independently of social-cognitive impairment. Together, these factors introduce potential sources of heterogeneity that further reduce certainty regarding the underlying mechanisms. Consequently, although current findings consistently point toward reduced contagious yawning, confidence in disorder-specific conclusions remains lower than for ASD and psychopathic traits.

### 4.2 Neurobiological mechanisms and the human mirror neuron system

The neurobiological evidence reviewed in this study supports the involvement of distributed neural systems rather than a single mechanism underlying contagious yawning. Across neuroimaging and electrophysiological studies, exposure to yawning stimuli was associated with activation or modulation of brain regions implicated in action observation, self-referential processing, mental state attribution, and broader social cognition, including the inferior frontal gyrus, posterior cingulate cortex, precuneus, and ventromedial prefrontal cortex (5, 14, 16, 25). Collectively, these findings are consistent with the behavioral evidence presented throughout this review, suggesting that contagious yawning reflects the coordinated interaction of multiple social-cognitive processes.

The human mirror neuron system (hMNS) has received particular attention because of its established role in action observation and imitation (12, 13). Functional neuroimaging demonstrating inferior frontal gyrus activation (5) and electrophysiological evidence of mu suppression during yawning observation (15) provide preliminary and indirect evidence consistent with the possible involvement of neural systems associated with action observation, including the human mirror neuron system (hMNS). However, the current evidence does not justify interpreting the hMNS as the primary mechanism underlying contagious yawning or its reduction across psychiatric disorders.

Several factors warrant caution. First, the available neurobiological studies are relatively few and generally involve small samples, limiting statistical power and reducing confidence in the reproducibility of individual findings. Second, the neural regions implicated in contagious yawning are not specific to this behavior but are also involved in multiple domains of social cognition, including empathy, mentalizing, self-other distinction, and action understanding.

Finally, the available neurobiological studies were generally limited by small samples and by the use of experimental paradigms that primarily assessed neural responses during exposure to yawning stimuli. These findings therefore provide evidence of neural involvement but do not establish that the observed neural activity causes contagious yawning behavior. Consequently, current neurobiological evidence should be viewed as complementary to the behavioral literature rather than as definitive support for any single mechanistic account.

### 4.3 Methodological considerations and limitations

The methodological appraisal identified variation in methodological characteristics across the included studies, with strengths and limitations differing according to study design.

Methodological appraisal should be distinguished from certainty of evidence, as differences in study design, sample characteristics, outcome measurement, and the directness of the evidence influence the strength of the conclusions that can be drawn.

First, the included studies differed in their capacity to address associations versus experimental effects. Several studies used observational or analytical cross-sectional approaches, whereas others employed quasi-experimental or randomized designs. Accordingly, the strength of causal inference varied across the evidence base, and the observed associations should be interpreted in relation to the design of each individual study.

Second, substantial methodological heterogeneity was evident across the reviewed literature. Studies differed in participant characteristics, diagnostic definitions, symptom severity, experimental paradigms, stimulus modality, familiarity manipulations, eye-fixation instructions, and outcome measures used to quantify contagious yawning. Such methodological variation likely contributes to the inconsistencies reported across studies by engaging partially distinct social-cognitive processes. Consequently, differences between studies should not necessarily be interpreted as conflicting evidence but rather as reflecting variation in experimental design and study populations.

Importantly, the strength of evidence differed across the mechanisms evaluated in this review. Behavioral evidence supporting the involvement of social attention and interpersonal processing was derived from multiple independent studies, including studies with relatively larger samples and controlled experimental manipulations.

In contrast, evidence supporting specific neurobiological mechanisms was based on comparatively few mechanistic investigations, many involving small sample sizes and exploratory analyses. Accordingly, confidence is currently greater for behavioral models of contagious yawning than for specific neurobiological explanations.

The certainty of evidence also differed across psychiatric conditions. ASD was represented by the largest and most methodologically consistent body of literature, allowing greater confidence in the reproducibility of the observed behavioral findings. By comparison, evidence regarding psychopathic traits was supported by fewer studies conducted primarily in non-clinical populations, while conclusions regarding schizophrenia-spectrum disorders remain tentative because only two eligible studies were identified. These differences should be considered when interpreting disorder-specific conclusions.

Finally, publication bias cannot be excluded. Given the relatively limited number of studies within several psychiatric populations, particularly schizophrenia-spectrum disorders, it remains possible that negative or null findings are underrepresented in the published literature. Furthermore, the considerable heterogeneity in study methodology and outcome measures precluded quantitative synthesis. Future research would benefit from greater standardization of contagious yawning paradigms, consistent diagnostic criteria, and harmonized outcome measures, facilitating more direct comparison across studies and enabling future meta-analytic investigations.

### 4.4 Future directions

Future research should examine contagious yawning as a multidimensional marker of social cognitive processing rather than as a direct measure of empathy. Studies incorporating simultaneous assessments of empathy, ToM, social attention, gaze behavior, emotional responsiveness, and interpersonal bonding may help clarify the relative contribution of these mechanisms. In ASD, combining contagious yawning paradigms with eye-tracking and biological measures such as oxytocin may help determine how social attention and biological heterogeneity influence contagious yawning susceptibility. In psychopathy, future studies should include clinically characterized populations and distinguish affective-interpersonal traits from broader aspects of social cognition. For schizophrenia-spectrum conditions, future research should further examine the contribution of social cognition impairments, negative symptoms, and medication effects while clearly distinguishing schizotypal traits from diagnosed schizophrenia. Finally, larger neuroimaging and electrophysiological studies using standardized paradigms are needed to clarify the neural basis of contagious yawning. Future work should consider interactions among action observation networks, the default mode network, and other systems involved in social cognition and self-other processing, rather than focusing exclusively on the human mirror neuron system.

### 4.5 Conclusions

This systematic review synthesized the available evidence regarding contagious yawning across Autism Spectrum Disorder, psychopathic traits, and schizophrenia-spectrum disorders while critically evaluating the mechanisms proposed to explain reduced contagious yawning. Across these psychiatric conditions, reduced contagious yawning emerged as a relatively consistent behavioral finding. However, the present review indicates that this phenomenon cannot be adequately explained by a single theoretical framework.

Overall, the available evidence most strongly supports interpreting contagious yawning as a multidimensional marker of social-cognitive functioning rather than as a direct behavioral measure of empathy. Although empathy may contribute to contagious yawning, the findings reviewed here suggest that social attention, interpersonal processing, emotional responsiveness, mental state attribution, and action observation all contribute to yawning susceptibility, with their relative importance varying across psychiatric conditions. Rather than representing competing explanations, these mechanisms are more appropriately viewed as complementary processes operating within broader social-cognitive networks.

Importantly, the strength of evidence is not equivalent across the proposed mechanisms.

Behavioral evidence examining social attention and interpersonal processing is supported by multiple independent studies employing diverse experimental paradigms. By contrast, evidence supporting specific neurobiological mechanisms remains preliminary because it is derived primarily from a limited number of mechanistic studies with relatively small samples.

Although the methodological appraisal identified methodological strengths across the reviewed studies, considerable heterogeneity in study design, participant characteristics, and contagious yawning paradigms limits direct comparison across investigations and reduces certainty regarding disorder-specific mechanisms.

Future research should therefore prioritize standardized experimental paradigms, adequately powered clinical samples, longitudinal designs, and multimodal behavioral and neurobiological approaches.

A clearer understanding of these mechanisms may ultimately determine whether contagious yawning represents merely an experimental phenomenon or a clinically informative behavioral marker of social-cognitive functioning across psychiatric disorders.
