# Supplementary material for: Reduced contagious yawning in psychiatric disorders: a systematic review of potential social-cognitive mechanisms
Source: Front Psychiatry. 2026 Oct 5;17:1879203. doi: 10.3389/fpsyt.2026.1879203 (PMC13677822; doi:10.3389/fpsyt.2026.1879203)
Supplement: Supplementary file 1 [file SupplementaryFile1.docx]

**Supplementary** **file 1. Search Strings**

The following are the search loops that were used in the various datasets:

**PubMed**

("Yawning"[Mesh]

OR "contagious yawning" [Title/Abstract]

OR "yawn contagion" [Title/Abstract]

OR (contagious [Title/Abstract] AND yawn*[Title/Abstract]))

AND

("Psychotic Disorders" [Mesh]

OR "Schizophrenia" [Mesh]

OR "Autism Spectrum Disorder" [Mesh]

OR "Antisocial Personality Disorder"[ Mesh]

OR schizo* [Title/Abstract]

OR psychot* [Title/Abstract]

OR psychopath* [Title/Abstract]

OR autis* [Title/Abstract]

OR "Autism Spectrum Disorder" [Title/Abstract]

OR "antisocial personality disorder" [Title/Abstract]

OR "callous-unemotional" [Title/Abstract])

**SCOPUS**

"contagious yawning" OR "yawn contagion" OR ( contagious AND yawn* ) OR yawn* AND schizo* OR psychot* OR psychopath* OR autis* OR "Autism Spectrum Disorder" OR "antisocial personality disorder" OR "callous unemotional"

**PsycINFO**

 (**Title**: "contagious yawning" *OR* **Title**: "yawn contagion") *OR* (**Abstract**: "contagious yawning" *OR* **Abstract**: "yawn contagion") *OR* (**Title**: yawn*) *OR* (**Abstract**: yawn*) *AND* **Any Field**: TI (schizo* OR psychot* OR psychopath* OR autis* OR "Autism Spectrum Disorder" OR "callous unemotional") *AND* **Abstract**: TI (schizo* OR psychot* OR psychopath* OR autis* OR "Autism Spectrum Disorder" OR "callous unemotional")
