# Supplementary material for: Reduced contagious yawning in psychiatric disorders: a systematic review of potential social-cognitive mechanisms
Source: Front Psychiatry. 2026 Oct 5;17:1879203. doi: 10.3389/fpsyt.2026.1879203 (PMC13677822; doi:10.3389/fpsyt.2026.1879203)
Supplement: Supplementary file 2 [file SupplementaryFile2.docx]

**Supplementary Table 1. Risk of bias assessment- The Joanna Briggs Institute (JBI) Critical Appraisal Checklist for analytical cross-sectional studies**

| **Study** | **Were the criteria for inclusion in the sample clearly defined?** | **Were the study subjects and the setting described in detail?** | **Was the exposure measured in a valid and reliable way?** | **Were objective, standard criteria used for measurement of the condition?** | **Were confounding factors identified?** | **Were strategies to deal with confounding factors stated?** | **Were the outcomes measured in a valid and reliable way?** | **Was appropriate statistical analysis used?** | **Overall appraisal** |
| --- | --- | --- | --- | --- | --- | --- | --- | --- | --- |
| Gallup et al., 2021 | Y | Y | Y | NA | Y | Y | U | Y | Included |
| Norscia et al., 2020 | Y | Y | Y | NA | Y | Y | Y | Y | Included |
| Bartholomew & Cirulli, 2014 | Y | Y | Y | NA | Y | Y | U | Y | Included |
| Norscia & Palagi, 2011 | Y | Y | Y | NA | Y | Y | Y | Y | Included |

**Supplementary Table 2. The Joanna Briggs Institute (JBI) Critical Appraisal Checklist for quasi-experimental studies**

| **Study** | **Q1** | **Q2** | **Q3** | **Q4** | **Q5** | **Q6** | **Q7** | **Q8** | **Q9** | **Overall appraisal** |
| --- | --- | --- | --- | --- | --- | --- | --- | --- | --- | --- |
| Poole & Henderson, 2023 | Y | N | NA | NA | Y | Y | Y | NA | Y | Included |
| Helt et al., 2021 — autistic vs. psychopathic traits | Y | N | NA | NA | NA | Y | Y | NA | Y | Included |
| Helt et al., 2021 — contagious itching | Y | Y | Y | Y | NA | Y | Y | NA | Y | Included |
| Helt et al., 2020 | Y | Y | Y | Y | NA | Y | Y | NA | Y | Included |
| Mariscal et al., 2019 | Y | Y | N | Y | NA | Y | Y | NA | Y | Included |
| Franzen et al., 2018 — Study 1 | Y | N | NA | NA | NA | Y | Y | NA | Y | Included |
| Chan & Tseng, 2017 | Y | N | NA | NA | NA | Y | Y | NA | Y | Included |
| Rundle et al., 2015 | Y | N | NA | NA | NA | Y | Y | NA | Y | Included |
| Haker et al., 2012/2013 | Y | N | NA | NA | NA | Y | Y | NA | Y | Included |
| Cooper et al., 2012 | Y | N | NA | NA | NA | Y | Y | U | Y | Included |
| Helt et al., 2010 — Study 2 | Y | Y | Y | Y | NA | Y | Y | NA | Y | Included |
| Helt et al., 2010 — Study 1 | Y | N | NA | NA | NA | Y | Y | NA | Y | Included |
| Arnott et al., 2009 | Y | N | NA | NA | NA | Y | Y | NA | Y | Included |
| Senju et al., 2009 | Y | Y | N | Y | NA | Y | Y | NA | Y | Included |
| Giganti & Esposito Ziello, 2009 | Y | Y | N | Y | NA | Y | Y | NA | U | Included |
| Haker & Rössler, 2009 | Y | Y | N | Y | NA | Y | Y | NA | Y | Included |
| Nahab et al., 2009 | Y | N | NA | NA | NA | Y | Y | NA | Y | Included |
| Senju et al., 2007 | Y | Y | N | Y | NA | Y | Y | NA | Y | Included |
| Platek et al., 2005 | Y | N | NA | NA | NA | Y | Y | NA | Y | Included |
| Platek et al., 2003 | Y | N | NA | NA | NA | Y | Y | NA | Y | Included |

**Quasi-experimental checklist questions:** Q1 Is it clear what is the cause and what is the effect? Q2 Was there a control group? Q3 Were participants included in comparisons similar? Q4 Were participants receiving similar treatment/care other than the exposure/intervention? Q5 Were there multiple measurements of the outcome both before and after the intervention/exposure? Q6 Were outcomes measured in the same way for the participants included in comparisons? Q7 Were outcomes measured in a reliable way? Q8 Was follow-up complete and, if not, were differences adequately described and analyzed? Q9 Was appropriate statistical analysis used.

**Supplementary Table 3. The Joanna Briggs Institute (JBI) Critical Appraisal Checklist for randomized controlled trials**

| **Study** | **Q1** | **Q2** | **Q3** | **Q4** | **Q5** | **Q6** | **Q7** | **Q8** | **Q9** | **Q10** | **Q11** | **Q12** | **Q13** | **Overall appraisal** |
| --- | --- | --- | --- | --- | --- | --- | --- | --- | --- | --- | --- | --- | --- | --- |
| Franzen et al., 2018 — Study 2 | Y | U | Y | U | U | Y | Y | Y | Y | Y | Y | Y | Y | Included |

**RCT checklist questions:** Q1 Was true randomization used for assignment of participants to treatment groups? Q2 Was allocation to treatment groups concealed? Q3 Were treatment groups similar at the baseline? Q4 Were participants blind to treatment assignment? Q5 Were those delivering treatment blind to treatment assignment? Q6 Were outcomes assessors blind to treatment assignment? Q7 Were treatment groups treated identically other than the intervention of interest? Q8 Was follow-up complete and, if not, were differences adequately described and analyzed? Q9 Were participants analyzed in the groups to which they were randomized? Q10 Were outcomes measured in the same way for treatment groups? Q11 Were outcomes measured in a reliable way? Q12 Was appropriate statistical analysis used? Q13 Was the trial design appropriate, and were any deviations from the standard RCT design accounted for in the conduct and analysis?

Y = Yes; N = No; U = Unclear; NA = Not applicable.
